# Hydrothermal-Freeze-Casting of Poly(amidoamine)-Modified Graphene Aerogels towards CO_2_ Adsorption

**DOI:** 10.3390/ijms22179333

**Published:** 2021-08-28

**Authors:** Alina Pruna, Alfonso Cárcel, Adolfo Benedito, Enrique Giménez

**Affiliations:** 1 Instituto de Tecnología de Materiales, Universitat Politècnica de València (UPV), s/n Camino de Vera, 46022 Valencia, Spain; acarcel@mcm.upv.es; 2 Instituto Tecnológico del Plástico (AIMPLAS), 4 Gustave Eiffel, Paterna, 46980 Valencia, Spain; abenedito@aimplas.es

**Keywords:** dendrimer, graphene oxide, hydrothermal synthesis, aerogel, adsorption

## Abstract

This article presents novel poly(amidoamine) (PAMAM) dendrimer-modified with partially-reduced graphene oxide (rGO) aerogels, obtained using the combined solvothermal synthesis-freeze-casting approach. The properties of modified aerogels are investigated with varying synthesis conditions, such as dendrimer generation (G), GO:PAMAM wt. ratio, solvothermal temperature, and freeze-casting rate. Scanning electron microscopy, Fourier Transform Infrared spectroscopy, Raman spectroscopy, X-ray photoelectron spectroscopy are employed to characterize the aerogels. The results indicate a strong correlation of the synthesis conditions with N content, N/C ratio, and nitrogen contributions in the modified aerogels. Our results show that the best CO_2_ adsorption performance was exhibited by the aerogels modified with higher generation (G7) dendrimer at low GO:PAMAM ratio as 2:0.1 mg mL^−1^ and obtained at higher solvothermal temperature and freeze-casting in liquid nitrogen. The enclosed results are indicative of a viable approach to modify graphene aerogels towards improving the CO_2_ capture.

## 1. Introduction

Although vital for our society, the energy from fossil fuels also comes with an important drawback due to CO_2_ emissions, which are considered to have the largest impact on global warming and climate change. The development of carbon capture technologies is seen as an effective way to mitigate such significant issues [1], and many alternatives have been for such purposes [2,3]. Amongst these, the development of hybrid solid sorbents based on porous materials with a high surface area, high pore volume adsorbent, and high adsorption and desorption rates is still a challenging task.

Carbon nanomaterials are widely studied thanks to their preferential CO_2_ sorption. Amongst these nanomaterials, graphene oxide (GO) offers high specific surface area, low-cost alternative, and tunable surface chemistry for low-temperature CO_2_ capture, thanks to the decorating hydroxyl and epoxide groups on the basal GO planes and carbonyl and carboxyl groups at the GO sheet edges [4].

The three-dimensional (3D) porous aerogels obtained from reduced graphene oxide (rGO) are considered promising sorbents for CO_2_ capture given their low density, interconnected porous network, large surface area, and active sites, which in turn are dependent on the synthesis route [5,6]. A two-step method based on simultaneous solvothermal reduction and self-assembly of GO and a subsequent freeze-drying procedure has been shown as a convenient approach for fabricating 3D GO-based aerogels [5,7,8,9,10,11,12,13]. Varying solvothermal conditions have been studied for the gelation and self-assembly of GO, such as temperature, duration, GO concentration, or GO flake size. 

Since the capture properties of aerogel sorbents are greatly dependent on their porosity, the freeze-casting method was proposed for templating well-defined porous structures by subsequent drying of frozen solvent crystals [14]. It was claimed that the freezing step is rather more important than the drying as the pores mirror the frozen solvent crystals [15]. Thus, the size and shape of the crystals depend on the cooling rate [16], the extent of freezing temperature, and solvent characteristics. It was reported that micropores become denser, and the pore size decreases as the temperature of the cooling source decreases. Enhanced properties of graphene aerogels, including thermal, electrical, and compressive ones, were obtained by decreasing the freezing temperature from −10 to −196 °C [17].

The freeze-drying techniques are usually adopted to avoid the restacking process of GO sheets upon partial reduction and result in high-surface area 3D structures [18]. However, the rGO is still decorated by residual oxygen-containing functional groups. The chemical modification or functionalization of GO nanosheets [19] by exploiting their oxygen functionalities as anchoring sites is considered a promising option—both to tune their properties and to overcome the aggregation of GO nanosheets that occurs when assembling GO nanomaterials into hierarchical architectures of interconnected sheets.

Small or large molecules containing amine groups have been reported for chemical modification of epoxide and carboxylic groups in GO to tailor its surface chemistry towards improved adsorption properties [10]. In this respect, poly(amidoamine) (PAMAM) dendrimers have attracted interest as they are non-volatile molecules with a high density of functional amine end groups and large surface area, thus providing an increased number of active sites, which are requisites for producing structures with improved performance [20]. The number of functional amine groups on the surface of PAMAM dendrimer increases exponentially with the generation (G) of dendrimer. PAMAM dendrimers represent a favorite alternative in several fields, such as electrochemical sensing or adsorption applications, due to their relatively low production cost, biodegradability [21], and unique structural properties, such as surface chemistry, controllable size and structure, hydrophilicity, and chemical stability [22,23,24].

Although there are many controversies regarding the distribution of the terminal end groups and their location on the surface of the molecule [25], which affect their surface reactivity, the amine groups in PAMAM dendrimers present an interest in CO_2_ capture [26]. The primary amine groups in PAMAM were indicated to react strongly with CO_2_ to produce a carbamate ion and a protonated base, while the tertiary amine group in PAMAM is not supposed to react directly with CO_2_ like a primary and secondary amine because they take the proton needed in the deprotonation step [27]. Kovvali and Sirkar were the first to report carbon capture on G0 PAMAM dendrimer immobilized in the pores of a hydrophilized microporous polymeric membrane [28]. Cross-linked poly(ethylene glycol) dimethacrylate (PEGDMA) polymer matrix with immobilized PAMAM showed CO_2_/H_2_ selectivity of 30 at 40 °C [29]. The amount of CO_2_ that was adsorbed also increased upon loading of amine-terminated G4 PAMAM dendrimer on cation-exchange clays [30]. CO_2_ adsorption was also studied on a sequence of generations from 1 to 7 of PAMAM-intercalated talc [31].

Under the above considerations, the combination of PAMAM dendrimers and GO nanomaterials has been proposed to develop hybrid structures with application in various fields, such as electrode applications or removing heavy metal ions from wastewater [32,33,34]. However, there are few studies on PAMAM-modified GO structures for CO_2_ capture.

This work reports a facile method to fabricate rGO aerogels modified with PAMAM dendrimers by solvothermal process towards CO_2_ adsorption. Moreover, the effect of freeze-casting rate on the formation of porous architectures is investigated. Since the cross-linking between GO and PAMAM and CO_2_ binding sites in PAMAM (nitrogen-containing groups) increase with the generation of dendrimer, it is of interest to investigate the adsorption capacity as a function of dendrimer generation and GO:PAMAM ratio. To the best of our knowledge, the effect of cross-linking between PAMAM and GO and freeze-casting of the corresponding hydrogels to form 3D hierarchical architectures has not been reported yet. Our combined hydrothermal-freeze-casting approach uses hydrogels of partially-reduced GO, obtained using hydrothermal synthesis. This novel approach is sought to afford rGO aerogels of improved potential for environmental remediation.

## 2. Results and Discussion

**Figure 1:**
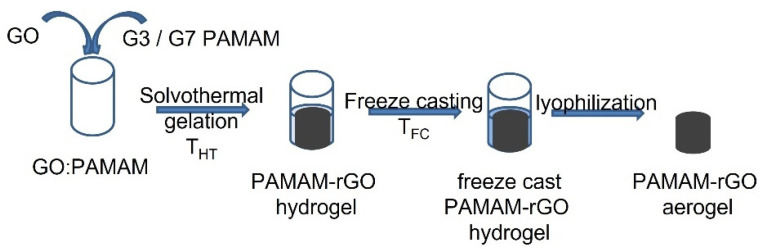
Scheme of the synthesis procedure.

This study investigated the solvothermal gelation of PAMAM modified GO-based hydrogels with the applied temperature, T_HT_, from 110 to 140 °C. The cross-linking of GO with PAMAM was also investigated with the dendrimer generation—namely, generation 3 (G3) and generation 7 (G7), and the GO:PAMAM wt. ratio from 1:0.1 to 1:0.6 (mg mL^−1^). The structure of the aerogels was tuned by varying the freeze-casting rate, and the resulting hydrogels were subjected to a cooling environment of temperature T_FC_ ranging from −5 to −196 °C. The modified aerogels were obtained upon lyophilization of the freeze-cast hydrogels. The effect of synthesis parameters on the adsorption performance was investigated at 298 K. The schematics of the synthesis procedure are presented in Figure 1.

Dendrimer characteristics, including size, shape, and number of amine groups on the surface, are known to vary with generation. Thus, the hydrodynamic diameter of PAMAM dendrimer is known to increase approximately 1 nm as each generation varies, reaching about 3.6 nm and 8.1 nm for G3 and G7, while the shape of dendrimer changes from planar elliptical to spherical one, for G3 and G7, respectively [35]. Moreover, it is known that the dendrimer generations higher than 4 present voids that can be exploited for complex formation; however, starting with generation 7, this core is inaccessible [36]. Thus, for the sake of simplification of the functionalization mechanism, this work considered dendrimers without core effects. 

PAMAM-modified rGO hydrogels with a fixed concentration of GO of 2 mg mL^−1^ were prepared by adding a PAMAM solution into the aqueous GO suspension under stirring in varying concentrations from 0.1 to 0.6 mg ml^−1^ and further subjection to hydrothermal synthesis at 140 °C for 4 h. All hydrogel shapes and dimensions were controlled by fixed container shape and volume. A successful hydrogel formation was reported for dendrimer concentration ranging from 0.1 to 0.6 mg mL^−1^. A GO:PAMAM wt. ratio as low as 1:0.1 was employed, namely, a concentration of 0.2 mg mL^−1^ in the case of G3, in agreement with the literature reporting such value as critical for self-assembling of rGO sheets with PAMAM dendrimer (G4) [37], while for G7 dendrimer, the concentration could be lowered to 0.1 mg mL^−1^ due to faster cross-linking as more amine groups decorate the dendrimer surface.

**Figure 2:**
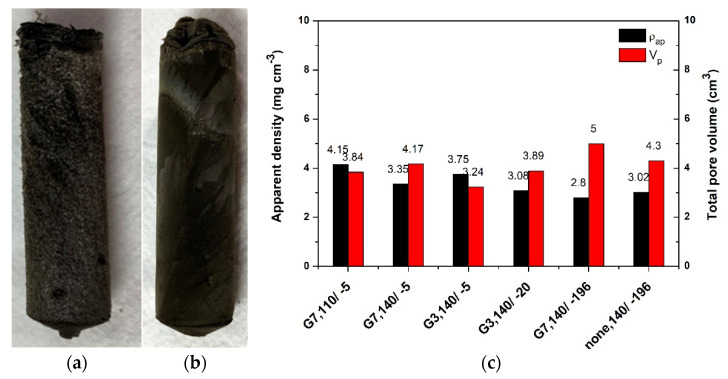
(**a**) Images of G7 PAMAM-modified rGO aerogels freeze-cast at T_FC_ of −5 °C and (**b**) −196 °C; (**c**) Evolution of apparent density and total pore volume (dendrimer concentration when present: 0.2 mg mL^−1^, T_HT_/T_FC_ °C).

The hydrogels were further subjected to varying freeze-casting and subsequently freeze-dried (lyophilized). While gelation resulted in a similar appearance, the visual inspection of the corresponding aerogel surface obtained upon lyophilization revealed differences as a function of synthesis parameters. For example, Figure 2a,b depict the typical PAMAM-modified rGO aerogels freeze-cast at T_FC_ of −5 °C and −196 °C, exemplified for the dendrimer G7 in a GO:PAMAM wt. ratio of 2:0.1 mg mL^−1^. As it can be observed, the modified aerogels show a well-consolidated 3D structure. Lower T_FC_ (higher freeze-casting rate) results in more compact monoliths with small pores and improved mechanical stability in comparison to the corresponding counterparts frozen at higher T_FC_, which is attributed to the smaller size of ice water crystals induced by the high freezing rate [17].

The effect of preparation parameters on the apparent density and pore volume was further analyzed. Representative values are shown in Figure 2c. The results show that the PAMAM-modified rGO aerogels exhibited ultra-low density varying from 2.8 to 4.15 mg cm^−3^. The density of PAMAM-modified rGO aerogels decreased with the increase in T_HT_ to 140 °C, due to improved reduction and modification with PAMAM of GO sheets, as suggested by comparing the rGO aerogel obtained in the absence and presence of PAMAM. By comparing the aerogels obtained from G7 and G3 generations of PAMAM dendrimer, it was observed that G7 resulted in lower density aerogels. This indicates a higher degree of reduction/incorporation of dendrimer that is in line with the increase in amine groups in this generation with respect to G3 one. Regarding the freeze-casting effect, lower T_FC_ was observed to lead to lower density aerogels for both generations, where the lowest density was exhibited by the aerogels freeze-cast in liquid nitrogen. These results are attributed to the homogeneous distribution of pores of smaller dimensions induced by instantaneous templating with liquid nitrogen.

On the other hand, the estimated pore volume results show that increasing T_HT_ results in higher pore volume suggesting a higher gelation rate. Using a dendrimer with a smaller number of primary amine groups (G3) resulted in lower pore volume. This indicates a lower cross-linking degree that agrees with the density evolution and can also be compared to the aerogel obtained with and without dendrimer freeze-cast in liquid nitrogen. The increase in the cooling rate resulted in higher pore volume for both dendrimer generations. This is due to the smaller size of solvent ice crystals induced by higher freeze-casting rate, resulting in a better-consolidated monolith with well-separated rGO sheets.

**Figure 3:**
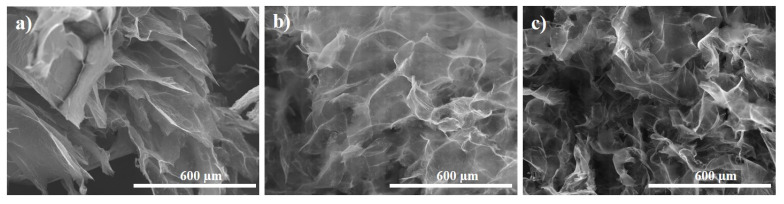
SEM images of modified rGO aerogels obtained: (**a**) In the absence and modified with (**b**) G3 and (**c**) G7 dendrimer.

The morphology of PAMAM-modified rGO aerogels was studied by SEM microscopy. First, a slow freeze-cast was applied (−5 °C), to study the effects of functionalization with dendrimer and gelation temperature, T_HT_. It was observed that the addition of dendrimer (GO:PAMAM 2:0.2 mg mL^−1^ ratio) generally resulted in interconnected porous microstructure with thinner rGO sheets, thus better separation degree, as demonstrated in Figure 3. The introduction of dendrimer resulted in improved distribution of thinner rGO sheets as evidenced by comparison with the size and opacity of rGO sheets in the non-modified aerogel that appears stacked in a non-homogeneous distribution. For a given concentration of dendrimer, thinner (more transparent) rGO sheets were observed in the resulting aerogels by modification with higher generation (G7) that is attributed to dendrimer size characteristics. It was reported that the cross-linking between the PAMAM dendrimers and rGO sheets proceeds between the amine groups at the surface of dendrimer and carboxylic groups in rGO [38]. Thus, in the case of G3, a planar molecule, the interaction would proceed horizontally from plane to plane. Whereas, in the case of the G7, the rGO sheets would be intercalated with globular dendrimer molecules of an increased size where the amine groups at the surface of the globular dendrimer are protonated by the carboxylic groups in rGO sheets, and thus, a more exfoliated structure is obtained [37].

**Figure 4:**
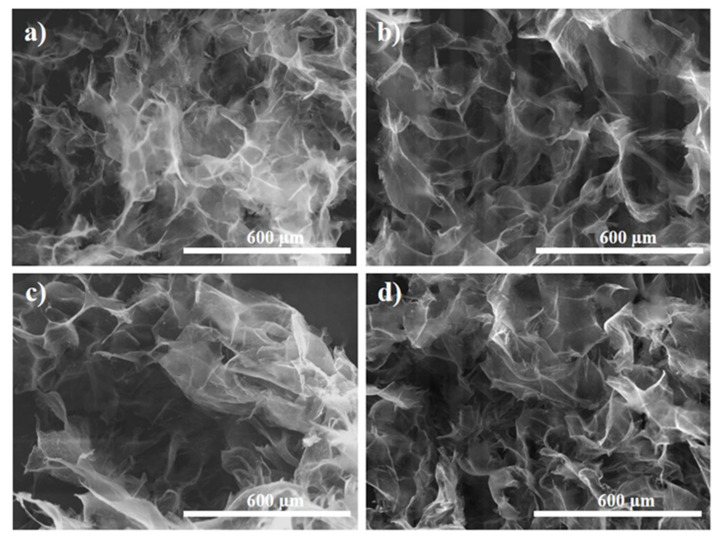
SEM images of PAMAM-modified rGO aerogels: G3 (0.6 mg mL^−1^) at T_HT_ of (**a**) 110 °C and (**b**) 140 °C and with G7 (0.2 mg mL^−1^) at T_HT_ of (**c**) 110 °C and (**d**) 140 °C.

The effect of T_HT_ was observed to be similar irrespectively of the dendrimer generation, as depicted in Figure 4. Thus, a lower temperature is observed to allow random higher rGO sheet stacking, indicating low functionalization degree and reduction rate. Whereas, by increasing the T_HT_ from 110 to 140 °C, the rGO sheets become thinner and better distributed, indicating improved functionalization. On the other hand, the pores in the aerogel are known to mirror the solvent ice crystals in the corresponding hydrogel upon freeze-drying, because the rGO sheets are repelled by ice crystals. Therefore, freeze-casting with liquid nitrogen generates a higher temperature gradient and instantaneous ice crystal formation with smaller size with respect to freeze-casting with lower T_FC_, such as that employed in a common freezer environment. This induces a predominant growth of the solvent crystal, with respect to the nucleation, and results in stacked rGO sheets along the ice crystals [39]. 

**Figure 5:**
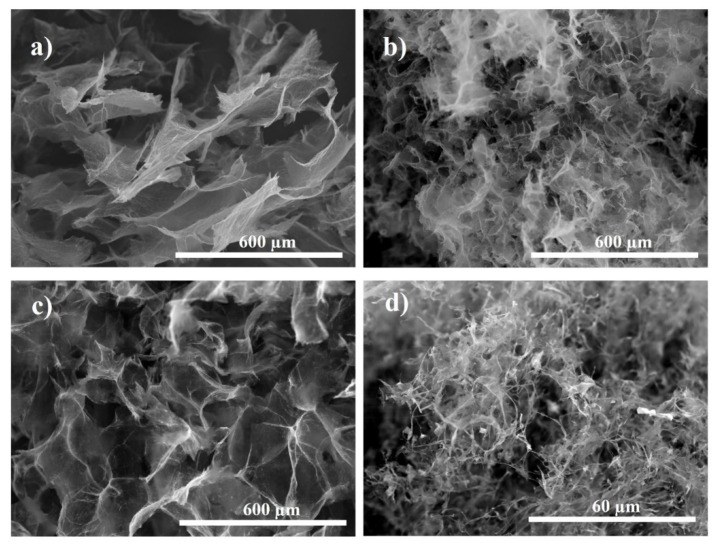
SEM images of un-modified rGO aerogels freeze-cast at: (**a**) −20 °C and (**b**) −196 °C and modified with G3 PAMAM freeze-cast at: (**c**) −5 °C and (**d**) −196 °C.

Further, the effect of T_FC_ was investigated. Figure 5a,b show the effects on the morphology of rGO aerogels obtained at T_HT_ of 140 °C as a function of T_FC_ in the absence of dendrimer. The results show that a lower freezing rate is detrimental for the homogeneous distribution of the rGO sheets and the pores, as the samples show randomly stacked rGO sheets (see Figure 3a and Figure 5a), which agrees with reported literature [14]. A higher freeze-casting rate (Figure 5b) resulted in more regular pattern of the pores in the aerogel, as well as homogeneously distributed rGO sheets of smaller size with a lower stacking degree than the ice water seeding became predominant. A combined effect of freeze-casting at high rate and dendrimer is further shown in Figure 5c,d. For exemplification, the images depict the rGO aerogels modified with G3 PAMAM at a concentration ratio 2:0.6 mg mL^−1^. It is clearly observed that upon addition of G3 dendrimer, the corresponding modified aerogel exhibits thinner rGO sheets and smaller, better distributed pores (see Figure 3a vs. Figure 5c for freeze-casting at −5 °C and Figure 5b vs. Figure 5d for freeze-casting at −196 °C). The highest freeze-casting rate (−196 °C) resulted in dendrimer-modified rGO aerogels with the thinnest rGO sheets and improved pore distribution, which also induced the best mechanical integrity.

**Figure 6:**
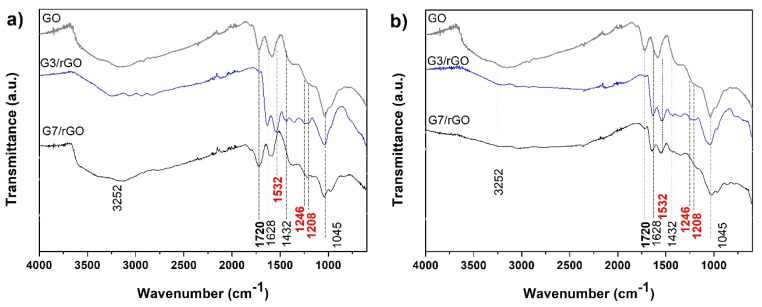
FTIR spectra of the PAMAM-modified rGO aerogels obtained at T_HT_ of (**a**) 110 °C and (**b**) 140 °C.

The typical FTIR spectra of PAMAM-modified rGO aerogels are depicted in Figure 6. The parent GO exhibited typical bands at about 1720 cm^−1^, 1610 cm^−1^, 1246 cm^−1^, and 1045 cm^−1^ and a wide band at 3200–3700 cm^−1^ attributed to carboxylic C=O, C=C, C–O–C, C–O, as well as O–H, respectively. Upon modification with dendrimer and formation of aerogel, the spectra showed less intercalated water molecules given that the corresponding wide band is less intense and the bands at 1720 cm^−1^ were absent, which is indicative of the lack of carboxylic groups. On the other hand, new peaks emerged at 1532 cm^−1^, which is characteristic for N–H vibration of amide groups, at 1246 and 1028 cm^−1^, which are attributed to C–N bonds and another vibration band at 1628 cm^−1^ assigned to amide C=O stretching vibration mode [40]. The new bands confirm the incorporation of PAMAM into the rGO aerogels by cross-linking between the carboxylic groups in GO and amine groups in PAMAM dendrimers [37,38,41,42,43]. By comparing the effect of dendrimer generation (primary amine group number) and T_HT_, it can be observed that above mentioned spectra modifications occur already at lower T_HT_ in the case of PAMAM G3, while G7 results in weaker modification of the bands. The difference is evident from the evolution of the band at 1720 cm^−1^ that disappears for G3 modified rGO aerogels, while it is still present in G7 modified ones. This indicates the carboxylic groups in GO interacted with the amine groups in the G3 dendrimer, while the interaction with those in G7 appeared absent. However, by increasing the T_HT_, both dendrimer generations result in modified FTIR bands, confirming their incorporation and indicate enhanced kinetics of amidation reaction with the synthesis temperature. This behavior could be explained by the access of the amine groups in the dendrimers for cross-linking with the carboxylic groups in GO, which is related to the characteristics of the dendrimers. Besides the size and shape effects, the number of amine groups on the surface is important in this regard as it increases exponentially with the generation (G3, G7), reaching 32 and 512 groups, respectively, which translates in a smaller distance between the functional groups and flexibility in G7 as compared to G3. It was reported that higher generations starting with G7 exhibit steric hindrance of the repetitive amide branches [44]. Therefore, the FTIR results suggest the planar shape and higher distance between the amine groups in the dendrimer G3 are favorable for amidation with carboxylic groups in GO also at lower T_HT_ due to easier accessibility, while for the spherical G7 dendrimer, a higher T_HT_ is needed to enhance the access of carboxylic groups in the GO sheets to the amines for amidation reaction.

XPS was further used to analyze the surface chemical nature of the PAMAM-modified rGO aerogels. The XPS results on N and O content, shown in Table 1, indicate that modification with a higher generation of dendrimer (G7) at increased T_HT_ results in increased N content both as wt.% and N/C ratio, which could be attributed to the higher number of amine groups in the higher generation of dendrimer and favorable interaction dendrimer-GO at higher process temperature. On the other hand, this synthesis approach also results in lower oxygen content, indicating a higher degree of reduction, which agrees with FTIR results. The freeze-casting at low T_FC_ by using liquid nitrogen appears to result in the highest nitrogen content in the modified aerogel, most probably because the smaller ice water crystals formed at such rate do not disrupt the network between dendrimer and rGO formed during the hydrothermal process. Moreover, it was suggested that oversized solvent crystals could damage the GO sheets, as well [17]. 

A similar evolution of N content was observed by an elemental analysis, as presented in Table 1. In general, these values were higher than the ones from XPS measurements. Although low, the N content appears in line with other works reporting values around 3% [45,46]. The N content in terms of wt.% in the modified rGO aerogel increased with the dendrimer generation [37], which is attributed to the increased number of primary amine groups and interaction with GO, as well as with the T_HT_, in agreement with the FTIR results. The N density, expressed by the ratio N/C followed the same trend, reaching N/C values similar to the literature [47].

**Figure 7:**
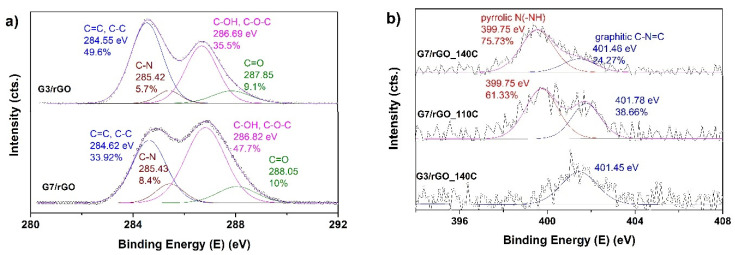
(**a**) High resolution XPS spectra of C1s peak for PAMAM-modified rGO aerogels obtained at 140 °C and (**b**) N1s peak for the PAMAM-modified rGO aerogels as a function of generation and T_HT_.

XPS high resolution spectra of C1s and N1s peaks are depicted in Figure 7 as a function of dendrimer generation and temperature of hydrothermal process. The deconvolution of C1s spectra of PAMAM-modified rGO aerogels depicted in Figure 4a showed the curves fit to a 4-peak component [40] with bonding energies at 284.55 eV, 285.42 eV, 286.7 eV, and 287.8 eV, respectively, being attributed to C=C species, C–N, C–O, and O=C–N species [48], which indicate that the PAMAM dendrimers were successfully grafted on the surface of rGO sheets. As dendrimer generation increased from 3 to 7, the contribution of O=C–N species increased. C–N also increased, due to the increased interaction of carboxylic groups in GO with amine groups in dendrimer. The successful introduction of dendrimers into rGO aerogels was also proved by the high resolution N1s spectra, as depicted in Figure 7b. It can be observed that the spectra of PAMAM-modified rGO aerogels were deconvoluted to one component, located at about 401.45 eV in the case of modification with G3 dendrimer, and two components, located at 399.75 eV and 401.46 eV in the case of G7. These are attributed to amine N bonds and amide N bonds, respectively [49]. The amine N is attributed to the amine groups in the PAMAM, and its contribution was observed with the increase in PAMAM generation, since G7 increased the number of amine groups. It was also found that the contribution to the total N content of amine N configuration increased with increasing T_HT_, in agreement with the FTIR results.

**Figure 8:**
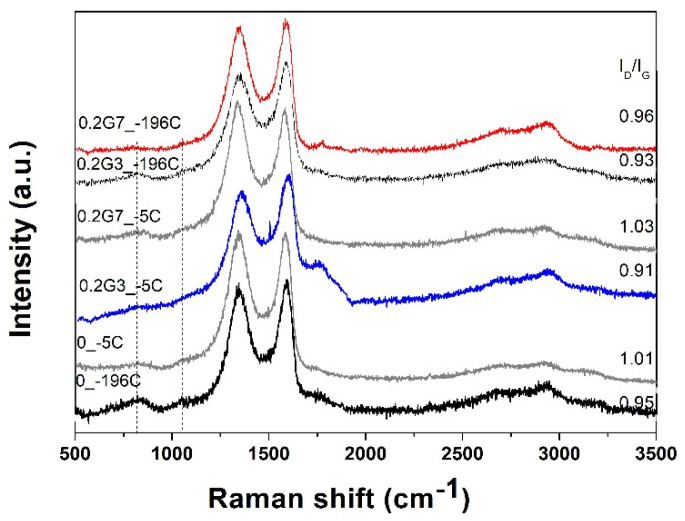
Raman spectra of dendrimer-modified rGO aerogels as a function of T_FC_ and generation.

Raman spectroscopy was further applied to investigate the electronic structure of the PAMAM-modified rGO aerogels. Typical spectra of dendrimer-modified rGO aerogels (0.2 mg mL^−1^) obtained at T_HT_ of 140 °C are depicted in Figure 8 as a function of dendrimer generation and TFC. All spectra were observed to exhibit the bands typical of GO, namely, a band located at about 1585 cm^−1^ (G band) ascribed to in-phase vibration of the E_2g_ mode of graphite lattice and the band located at about 1340 cm^−1^ (D band) ascribed to the defects in the rGO lattice and the edge effect of graphene crystallites [50]. The presence of dendrimer in the modified rGO aerogels is indicated by the evolution of the band located at about 1047 cm^−1^ present in all spectra, which represents a vibration due to coupling of skeletal stretching and amine modes [50]. For evaluating the disorder in the lattice and defects in modified rGO sheets, the intensity ratio between the D and G bands (I_D_/I_G_) is typically employed. Generally, it was observed that slow freeze-casting results in a higher intensity ratio, which is explained by the disruption of the rGO sheets, due to the formation of oversized solvent ice crystals [17]. The dendrimer molecules could serve as a spacer, meaning the disruption of the modified rGO sheets will have a less extent, and thus, a slightly lower intensity ratio is obtained upon modification with the G3 dendrimer molecules. On the other hand, the introduction of G7 dendrimer with increased amine groups leads to greater interaction with the carboxylic groups in rGO, resulting in a slightly higher intensity ratio. These effects are observed both at slow and fast freeze-casting. The difference in intensity ratio is much smaller at fast freeze-casting, since the almost instantaneously frozen, and thus, less defective rGO sheets are obtained. It is suggested that the defects in rGO lattice are governed by the formation of solvent crystals at a low freezing rate, while modification with dendrimer is more evident in the case of G7 molecules. This is based on the above observations and on a slight shift in G band for the fast freeze-casting of un-modified rGO aerogel (1593 cm^−1^) with respect to the dendrimer-modified one (1591 cm^−1^), due to the charge transfer between GO and PAMAM [37], while the interaction with dendrimer did not appear to affect the G band position in the case of low freeze-casting rate. 

The pore characteristics play an important role in developing a high-capacity porous adsorbent. Thus, to characterize porous structures with pore size within 100 nm, nitrogen adsorption/desorption analysis at 77 K is usually employed. According to the sorption isotherms, the porosity characteristics were calculated, and the results are listed in Table 2. The BET results are consistent with the FTIR results, internal nanostructure, and apparent density mentioned above. The SSA values calculated by the BET method were between 50 and 111 m^2^ g^−1^. The increase in T_HT_ resulted in higher SSA, being attributed to improved interaction between the dendrimer and rGO sheets. The increase in the freeze-casting rate (lower T_FC_) resulted in slightly decreased SSA, and it is attributed to the formation of small pores due to the increased number of smaller solvent ice crystals. However, almost 2-fold SSA was obtained with a lower dendrimer concentration, for both generations. This may indicate a steric hindrance of the amine groups at higher concentration values. At the same concentration, the modification with higher generation dendrimer resulted in smaller SSA, which might be explained by much smaller pores due to increased interaction. The highest values of total pore volume TV_p_ (0.15 cm^3^ g^−1^) were exhibited by the aerogels obtained at 140 °C, freeze-cast at low T_FC_, and containing a low concentration of dendrimer. 

Besides the BET analysis, the desorption branch of the nitrogen isotherm was analyzed with the BJH model for the mesopore surface area, and average pore diameter and volume. The BJH pore area was found higher than SSA. The mesopore surface area and pore volume followed a similar trend with the fabrication conditions as the SSA values, and they ranged from 113 to 194 m2 g^−1^ and from 0.132 to 0.306 cm^3^ g^−1^, respectively. The BJH results also indicated pores with diameters between 1.9 and 2.4 nm consistent with mesoporous characteristics (2 nm < diameter < 50 nm) in the PAMAM-modified rGO aerogels.

The DR model was used to complement the study on porosity with respect to micropores with the volume V_DR_, surface area SA_DR_, the characteristic energy of adsorption (E_0_, kJ mol^−1^). The results indicated a 10 times smaller micropore surface area with respect to mesopore surface area, while the micropore volume was even much lower than the correspondent mesopore volume. The highest energy of adsorption was obtained for the aerogels modified with a lower concentration of dendrimer, higher T_HT_, and freeze-cast at a fast rate—which agrees with the BET and BJH results.

**Figure 9:**
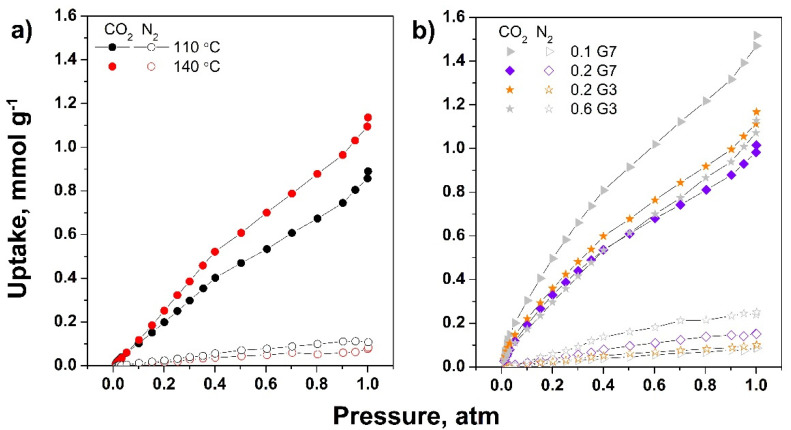
CO_2_ and N_2_ adsorption isotherms on dendrimer-modified rGO aerogels as function of T_HT_ (**a**) and on aerogels modified with varying dendrimer generation and concentration freeze-cast at −196 °C (**b**).

The adsorption capacity for both CO_2_ and N_2_ at 298 K of the dendrimer-modified rGO aerogels are further shown in Figure 9. The effect of T_HT_ on the uptake of G7-modified rGO aerogels (0.2 mg mL^−1^, freeze-cast at −5 °C), depicted in Figure 9a, shows an increase in CO_2_ uptake with T_HT_. Moreover, the low adsorption of N_2_ was exhibited in both cases and the adsorption selectivity for CO_2_ over N_2_ was observed to increase with T_HT_ from 9 to 21. These results may be attributed to increased functionalization and N content with T_HT_, in agreement with FTIR, XPS, and EDS results. The modified aerogels freeze-cast at varying T_FC_ (not shown) exhibited CO_2_ uptake ranging from 1.14 to 1.02 mmol g^−1^, slightly decreasing with T_FC_, which are attributed to the pore characteristics shown in Table 2. However, given the improved mechanical stability and increased N/C ratio, and lower defect degree obtained by freeze-casting at high T_FC_, the highest freezing rate was further selected to investigate the effect of dendrimer. Thus, Figure 9b shows the effect of concentration and generation of dendrimer over the selective adsorption of CO_2_ over N_2_ on the modified aerogels obtained at T_HT_ of 140 °C and T_FC_ of −196 °C.

It was observed that the high concentration of G7 dendrimer exhibited lower CO_2_ uptake of 1.02 mmol g^−1^, which could be due to lower SSA, SA_BJH_, and low TV_p_, as depicted in Table 2. The lower efficiency may also be attributed to lower accessibility to active sites as the terminal primary amine groups are close to the dendrimer core, and thus, they are unavailable for adsorption [31]. On this premise, it was observed that by using a dendrimer with a lower number of amine groups, thus with reduced steric hindrance, the CO_2_ uptake increases, in agreement with the increase in SSA, SA_BJH_, and TV_p_. A higher concentration of dendrimer is detrimental to further improvement in performance, most probably due to stacking and lowered porosity, as shown in Table 2, or lower distribution of dendrimer in the aerogel network [26,51]. Although the pore characteristics did not improve any further, the best uptake performance (1.52 mmol g^−1^) was obtained at the lowest concentration of dendrimer of high generation, which is attributed to improved interaction between dendrimer and rGO. The number of amine groups increased, as supported by the N/C values depicted in Table 1, which also increased at lower dendrimer concentration of higher generation and by XPS results, indicating an increased contribution in amine N bonds at higher generation dendrimer. The selectivity for CO_2_ over N_2_ increased in the same trend, from 11 to 21 and 38 for the modified aerogels with 0.2 mg mL^−1^ G7, 0.2 mg mL^−1^ G3, and 0.1 mg mL^−1^ G7, respectively. These results indicate the adsorption performance was not only affected by pore characteristics, but also by N content in terms of N/C ratio and nitrogen binding contributions to total nitrogen content, as indicated in recent literature [7,8,10,52].

The obtained adsorption performance agreed the reported literature [53]. Although it is difficult to perform a straightforward comparison with available reports due to many parameters involved in the measurements, including the conditions for degassing (here, a temperature below 100 °C was applied so as not to induce a supplementary reduction of GO material), values between 1 and 1.5 mmol g^−1^ have been reported for other sorbents degassed in similar conditions [54,55,56].

## 3. Materials and Methods

The reagents (Alfa Aesar, Madrid, Spain) used in this study were employed as received. The GO aqueous slurry was provided by Graphenea SA (Donostia, Spain). Prior to use, the aqueous dispersion of GO flakes (2 mg mL^−1^) was obtained by ultrasound bath treatment for 1 h. Generation 3 and 7 of PAMAM dendrimers were purchased from Sigma Aldrich (Madrid, Spain) and kept in a common freezer until use. 

PAMAM dendrimer was added to GO dispersion in a concentration ranging from 0.1 to 0.6 mg mL^−1^. PAMAM generation 3 (G3) with 32 amine groups on the periphery and generation 7 (G7) with 512 peripheric amine groups were considered in the study for the effect of the number of amine groups. Upon 15 min sonication, the mixtures were subjected to gelation by solvothermal approach for 4 h at a temperature value ranging from 110 to 140 °C (temperature in solvothermal process, T_HT_). The simultaneous thermal reduction and cross-linking with dendrimer was applied to partially reduce the GO, and thus, to tune the C/O ratio in rGO. Cylindrical hydrogels were obtained with diameter and height controlled by the shape of the container. 

The as-prepared hydrogels were frozen under varying freezing rates by subjecting the containers to different cooling conditions ranging from −5 to −196 °C (freeze-casting temperature, T_FC_). The cooling conditions were achieved by using a common freezer, refrigerant mixture water/ethylene glycol, and liquid nitrogen. The duration of exposure to cold conditions was 48 h, except for the samples obtained by exposure to liquid nitrogen, which were frozen instantaneously. The frozen specimens were subjected to freeze-drying by sublimation at 20 °C for 48 h at 0.015 mbar to remove the ice water crystals and obtain the corresponding aerogels. No further reduction of aerogels was performed.

The apparent aerogel density was estimated by dividing the weight w of the aerogels by their volume V (triplicate measurements), and measured with a caliper (measurement error is estimated as ±10%). The total pore volume (V_p_) was calculated by using the relationship V_p_ = V−w/ρ_rGO_, where ρ_rGO_ is the density of rGO assumed as 1.06 g cm^−3^ [57]. The morphology of the aerogels (top, down, and middle section) was studied by using a scanning electron microscope (SEM, JSM 6300 JEOL, Tokyo, Japan). Energy Dispersive X-Ray Spectrometer (EDS, Oxford Instruments, Bristol, UK) was employed for elemental composition measurements. Flash combustion (average of three sample measurements) was employed to analyze the nitrogen content in the modified aerogels by using a Flash Smart Eager 200 (Thermo Scientific, Madrid, Spain) analyzer. The modification of the functional groups in rGO aerogels was assessed by means of Fourier-transform infrared spectroscopy (FTIR) in ATR mode in the spectral window of 4000–400 cm^−1^ with an FT/IR-6200 (Jasco, Madrid, Spain) spectrometer. The nitrogen contributions in the modified aerogels were studied by X-ray Photoelectron Spectroscopy (XPS) by using a photoelectron spectrometer VG-Microtech Multilab 3000 (Thermo Fisher Scientific Inc., Waltham, MA, USA). Curve deconvolution was performed using CASAXPS 2.3.17 software (Casa Software Ltd., Wilmslow, Cheshire, UK) by applying a Shirley baseline subtraction and a Gaussian–Lorentzian (70%:30%) peak shape. Raman spectra were acquired on an Xplora spectroscope employing a 532 nm laser (Horiba, Villeneuve d’Ascq, France). Nitrogen adsorption isotherms measured at 77 K on an ASAP 2420 analyzer (Micromeritics, Norcross, GA, USA) were performed for the specific surface areas (SSA) of PAMAM-modified rGO aerogels using the Brunauer–Emmett–Teller (BET) method. Total pore volume was measured at a relative pressure of 0.95 for pores below 50 nm diameter. The mesopore surface area (SA_BJH_) and mesopore volume (V_BJH_) were both analyzed by the Barrett–Joyner–Halenda (BJH) method, using the data of the desorption branch. Dubinin–Radushkevich (DR) model was applied for the analysis of the micropore surface area (SA_DR_) and micropore volume (V_DR_). Measurements were performed in triplicate.

To assess the adsorption performance of the PAMAM-modified rGO aerogels, the samples were first outgassed under vacuum at 80 °C for 24 h and further subjected to CO_2_ adsorption/desorption measurements at 298 K. The isotherms were recorded up to 1 bar on ASAP 2420 analyzer (Micromeritics, Norcross, GA, USA). Selective CO_2_ adsorption over N_2_ was determined by also measuring the N_2_ adsorption performance at 298 K. The selectivity was calculated as mentioned elsewhere [58].

## 4. Conclusions

PAMAM dendrimers were employed to modify rGO aerogels by a combined hydrothermal-freeze-casting approach. The properties of modified aerogels were studied with varying parameters (generation and concentration of dendrimer, hydrothermal synthesis temperature, and freeze-casting temperature). The results indicated improved functionalization with dendrimer and N/C ratio of the aerogels by increasing the hydrothermal temperature from 110 to 140 °C. The XPS results showed that the rGO aerogels that were modified with generation 7 dendrimer exhibited different nitrogen contributions with respect to those modified with generation 3—namely, pyrrolic, which was present beside the graphitic nitrogen contribution. By lowering the freeze-casting temperature from −5 to −196 °C, more homogeneous, better mechanically stable aerogels can be obtained, which also exhibit increasing N content attributed to lower disruption of the aerogel network. An increased concentration of dendrimer (0.2 mg mL^−1^ for generation 7 and 0.6 mg mL^−1^ for generation 3) was shown to detrimental improve adsorption performance, likely due to steric hindrance to active sites. The best performance, in terms of CO_2_ capture and selectivity over N_2_, was exhibited by the aerogel modified with higher generation at the lowest concentration—reaching the values of 1.52 mmol g^−1^ of adsorbed CO_2_ and 38 for selectivity Our results indicate that adsorption performance can be tailored by a proper balance of porosity and N/C in the aerogels.

## Figures and Tables

**Table 1:** Chemical composition wt.% by XPS measurements and elemental analysis. (sample notation: generation/mg mL^−1^ of dendrimer/T_HT_, °C/T_FC_, °C).

Sample	XPS Analysis			Elemental Analysis	
	N, %	N/C	O/C	N, %	N/C
G3/0.2/140/−5	0.29	0.004	0.43	0.54	0.01
G7/0.1/140/−5				1.6	0.033
G7/0.2/140/−196	4.45	0.066	0.41		
G7/0.2/140/−5	1.03	0.014	0.34	1.5	0.03
G7/0.2/110/−5	0.75	0.01	0.36	1.28	0.026

**Table 2:** Nitrogen adsorption results as a function of synthesis conditions. (sample notation: generation/mg mL^−1^ of dendrimer/T_HT_, °C/T_FC_, °C).

Sample	SSA m² g^−1^	TV_p_ cm^3^ g^−1^	SA_BJH_ m² g^−1^	V_BJH_ cm^3^ g^−1^	E_0_ kJ mol^−1^	V_DR_ cm^3^ g^−1^	SA_DR_ m² g^−1^
G7/0.2/110/−5	68.2	0.08	125	0.132	9.2	0.004	12.2
G7/0.2/140/−5	109	0.11	172	0.177	6.478	0.013	36.2
G7/0.1/140/−20	87.4	0.09	165	0.186	7.051	0.010	29.1
G7/0.2/140/−196	50.1	0.08	113	0.192	14.6	0.007	19.6
G7/0.1/140/−196	108.2	0.14	185	0.236	15.23	0.008	22.7
G3/0.6/140/−196	82	0.11	122	0.204	14.32	0.007	19.6
G3/0.2/140/−196	111	0.15	194	0.306	14.68	0.010	28.7
